# Low-arginine and low-protein diets induce hepatic lipid accumulation through different mechanisms in growing rats

**DOI:** 10.1186/s12986-020-00477-5

**Published:** 2020-08-03

**Authors:** Lila Otani, Hiroki Nishi, Ayaka Koyama, Yuta Akasaka, Yusuke Taguchi, Yuka Toyoshima, Daisuke Yamanaka, Fumihiko Hakuno, Huijuan Jia, Shin-Ichiro Takahashi, Hisanori Kato

**Affiliations:** 1 Department of Agricultural Biological Chemistry, The University of Tokyo, Bunkyo-ku, Tokyo, 113-8657 Japan; 2 Department of Animal Sciences, The University of Tokyo, Tokyo, Japan; 3 Department of Veterinary Medical Sciences, Graduate School of Agricultural and Life Sciences, The University of Tokyo, Tokyo, Japan; 4 Department of Bioregulation, Institute for Advanced Medical Sciences, Nippon Medical School, Tokyo, Japan

**Keywords:** Arginine deficiency, Low-protein diet, Hepatosteatosis, Apolipoprotein A-IV, Insulin signaling

## Abstract

**Background:**

Dietary protein deficiency and amino acid imbalance cause hepatic fat accumulation. We previously demonstrated that only arginine deficiency or total amino acid deficiency in a diet caused significant hepatic triglyceride (TG) accumulation in young Wistar rats. In this study, we explored the mechanisms of fatty liver formation in these models.

**Methods:**

We fed 6-week-old male Wistar rats a control diet (containing an amino acid mixture equivalent to 15% protein), a low-total-amino acid diet (equivalent to 5% protein; 5PAA), and a low-arginine diet (only the arginine content is as low as that of the 5PAA diet) for 2 weeks.

**Results:**

Much greater hepatic TG accumulation was observed in the low-arginine group than in the low-total-amino acid group. The lipid consumption rate and fatty acid uptake in the liver did not significantly differ between the groups. In contrast, the low-total-amino acid diet potentiated insulin sensitivity and related signaling in the liver and enhanced de novo lipogenesis. The low-arginine diet also inhibited hepatic very-low-density lipoprotein secretion without affecting hepatic insulin signaling and lipogenesis.

**Conclusions:**

Although the arginine content of the low-arginine diet was as low as that of the low-total-amino acid diet, the two diets caused fatty liver via completely different mechanisms. Enhanced lipogenesis was the primary cause of a low-protein diet-induced fatty liver, whereas lower very-low-density lipoprotein secretion caused low-arginine diet-induced fatty liver.

## Background

Dietary protein has a strong impact on growth, body composition, and energy homeostasis. Protein overload results in a reduced body fat in humans [1, 2] and in a improve insulin sensitivity in rats [3, 4]. By contrast, dietary protein malnutrition induces hepatic fat accumulation, a symptom of kwashiorkor, in rats [5] and humans [6]. The dietary amino acid balance also affects fat deposition in the liver. For example, rats fed low-protein diets supplemented with essential amino acids, such methionine, experience excessive fat deposition in the liver, and supplementation of a low-protein diet with DL-threonine prevents hepatic fat accumulation [7].

Non-alcoholic fatty liver disease (NAFLD) represents a series of hepatic disorders triggered by excessive liver fat accumulation and is associated with central obesity and insulin resistance. While the effects of dietary fat and carbohydrates on liver fat accumulation have been extensively studied, the effects of dietary protein [8, 9] and amino acids [10] on energy homeostasis are less well understood despite their importance in the pathogenesis of NAFLD. For example, insulin is a major anabolic hormone that is intimately involved in hepatic metabolism, and its secretion is affected by dietary protein [11] and amino acid intake [12]. Protein malnutrition suppresses insulin secretion from the pancreas in response to glucose intake [9, 13]. Some amino acids, such as arginine and leucine, are also known to stimulate insulin secretion [12, 14]. Moreover, our group previously demonstrated that dietary protein malnutrition decreased plasma insulin and insulin-like growth factor 1 concentrations [9, 15], and upregulated the protein expression of insulin receptor substrate 2 (IRS2), one of the key mediators of insulin signaling, in the livers of growing rats, resulting in increased insulin sensitivity [16]. These results implied that enhanced insulin sensitivity caused by dietary protein deficiency may mediate fatty liver development.

We previously investigated the impacts of various amino acids on fat accumulation in the liver using amino acid-deficient diets, and the results showed that the liver triglyceride (TG) content could be increased only by a deficiency of arginine out of the 20 major amino acids tested [10]. Therefore, we hypothesized that lipid accumulation in the liver induced by dietary amino acid deficiency could be attributed to increased insulin sensitivity, which may be caused specifically by dietary arginine deficiency. Here, we tested this hypothesis by evaluating metabolic status and hepatic lipid flux in models of total amino acid and arginine deficiency.

## Methods and materials

### Materials

Anti-insulin receptor subunit β (IRβ) and anti-CD36 antibodies for immunoblotting were purchased from Santa Cruz Biotechnology (Dallas, TX, USA). The anti-IRS2 (clone 9.5.2) antibody for immunoblotting was purchased from Merck Millipore (Billerica, MA, USA). Anti-phospho-p70 S6K (Thr389), anti-p70 S6K (49D7), anti-phospho-4E-BP1 (Thr37/46), anti-4E-BP1, and anti-phospho-AMPKα (Thr172) (40H9) antibodies were purchased from Cell Signaling Technology (Danvers, MA, USA). The anti-GAPDH (6C5) antibody was purchased from Abcam (Cambridge, UK). Anti-rabbit and mouse IgG horseradish peroxidase-conjugated secondary antibodies were purchased from GE Healthcare (Little Chalfont, UK).

### Animals

The experiments were approved by the Animal Usage Committee of the Faculty of Agriculture of the University of Tokyo and performed in accordance with its guidelines (Permission No. P09–375). Male Wistar rats were purchased from Charles River Laboratories International (Kanagawa, Japan). The animals were housed individually in wire cages with free access to food and water. The rats were maintained at a room temperature of 23 °C ± 1 °C with 50–60% relative humidity under a 12-h light/dark cycle (light from 08:00 to 20:00). In the pre-experimental period, the rats were fed a purified diet containing 15% protein from casein.

### Comparison of the effects of the low-total-amino acid and low-arginine diets

Six-week-old male Wistar rats were randomly divided into 3 groups with different diets: the amino acid mixture control diet (equivalent to 15% protein in the diet; 15PAA), low-arginine diet in which the concentration of arginine in young rodents was 33% of that in the 15PAA diet (low Arg, *n* = 8), and the low-total-amino acid diet (equivalent to 5% protein in the diet; 5PAA). The low-total-amino acid mixture simulated the composition of casein [17]. The diet compositions are shown in Tables 1 and 2. Rats were given ad libitum access to tap water and food. Body weight and food intake were measured daily. Fourteen days after initiation of the experimental diets, the rats were anesthetized by intraperitoneal injection of pentobarbital (30 mg/kg) 1 h after removal of the diet, and a postprandial blood sample was collected from the carotid artery. The liver, longissimus muscles, and abdominal fat were removed and weighed. The livers and muscle tissues were soaked in RNAlater (AMBION, Austin, TX, USA) or snap-frozen in liquid nitrogen and stored at − 80 °C until use. The organ samples were used to analyze tissue TG concentrations, and protein and RNA expression.

**Table 1:** Composition of diet

Component	15P AA	5PAA	Low Arg
	g/kg diet		
Amino acid mixture^a^	150.0	50.0	146.5
DL-methionine	2.5	0.8	2.5
Corn starch	620.5	747.2	649.0
Soy bean oil	50.0	50.0	50.0
Vitamin mixture^b^	10.0	10.0	10.0
Mineral mixture^b^	40.0	40.0	40.0
Choline chloride	2.0	2.0	2.0
Cellulose powder	100.0	100.0	100.0

^a^Composition of amino acid mixture were described in Table 2

^b^AIN-76 prescription (Oriental Yeast Co., ltd)

**Table 2:** The amino acid content of each diet

	15PAA	5PAA	Low Arg
	g/kg diet		
Alanine	4.08	**1.36**	4.08
Arginine	5.25	**1.75**	**1.75**
Asparagine-H_2_0	5.76	**1.92**	5.76
Aspartate	5.05	**1.68**	5.05
Cystine	0.8	**0.27**	0.80
Glutamate	14.65	**4.88**	14.65
Glutamine	14.65	**4.88**	14.65
Glycine	2.59	**0.86**	2.59
Histidine	4.06	**1.35**	4.06
Isoleucine	7.12	**2.37**	7.12
Leucine	13	**4.33**	13.00
Lysine-HCl	14.1	**4.70**	14.10
Methionine	3.89	**1.30**	3.89
Phenylalanine	7.2	**2.40**	7.20
Proline	14.98	**4.99**	14.98
Serine	8.09	**2.70**	8.09
Threonine	6.09	**2.03**	6.09
Tryptophan	7.76	**0.58**	1.73
Tyrosine	1.73	**2.59**	7.76
Valine	9.16	**3.05**	9.16
Total volume	150.00	50.00	146.50

We performed oral glucose tolerance tests (OGTTs) 12 d after initiation of the experimental diets. After 16 h of overnight fasting, glucose (2 g/kg) was orally administered to the rats. Blood (preprandial) was collected from the tail vein into heparinized tubes that were chilled on ice. The blood samples were subjected to centrifugation at 3000×*g* for 5 min at 4 °C and the supernatants were transferred to new tubes. Plasma samples were stored at − 80 °C until analysis.

### Blood biochemistry

Blood biochemical parameters, including total cholesterol, TG, free fatty acids (FFA), and glucose concentrations, were determined using commercial kits (Cholesterol E-test, Triglyceride E-test, NEFA C-test, and Glucose CII test, respectively; Wako Pure Chemical Industries, Osaka, Japan). The plasma insulin concentration was measured using an insulin measurement kit (Morinaga Institute of Biological Science, Yokohama, Japan) according to the manufacturer’s instructions.

### Lipid extraction and TG measurements

Lipids were extracted from frozen livers and longissimus muscles via modified Folch method [18] in a 2:1 (vol/vol) mixture of chloroform/methanol. The extracts were washed with 0.5 volumes of 0.8% KCl and centrifuged at 1500×*g* for 10 min, and the organic phases were recovered. The TG content in the liver and plasma was also determined using a commercial kit (Wako Pure Chemical Industries) according to the manufacturer’s instructions.

### RNA extraction and reverse transcription-polymerase chain reaction

Total RNA was isolated from homogenized livers using NucleoSpin® RNA (Macherey-Nagel, Düren, Germany) according to the manufacturer’s instructions. The total RNA concentration was measured with a NanoDrop® spectrophotometer (ND-1000, NanoDrop, Wilmington, DE, USA). The quality of the RNA was determined by assessing the A260/280 ratio and by agarose gel electrophoresis. The RNA was reverse-transcribed into cDNA using PrimeScript® RT Master Mix (Takara Bio, Shiga, Japan). cDNA was amplified using SYBR® Premix Ex Taq II (Takara Bio) according to the manufacturer’s protocol. We designed the primers for reverse transcription (RT) polymerase chain reaction (PCR) with the design software Primer 3. β-actin was used as an endogenous control. The following PCR primers were used: β-actin (*Actb*) forward, 5′-GGAGATTACTGCCCTGGCTCCTA-3′, and reverse, 5′-GACTCATCGTACTCCTGCTTGCTG-3′; MTP (*Mttp*) forward, 5′-AGCAACATGCCTACTTCTTACAC-3′, and reverse, 5′-TCACGGGTTCACTTTCACTG-3′; apolipoprotein A-IV (*ApoA4* or *Apoa4*) forward, 5′-ACCCTCTTCCAGGACAAACTTG-3′, and reverse, 5′-CCTTGGTTAGATGTCCACTCAGTTG-3′; and apolipoprotein B (*ApoB* or *Apob*) forward, 5′-CCTGTCCATTCAAAACTACCACA-3′, and reverse 5′-CAATGAACGAATCAGAAGGTGA-3′.

### Western blotting

Western blotting analysis was performed as previously described [9, 16]. In brief, frozen livers were homogenized in homogenizing buffer and centrifuged at 100,000×*g* for 1 h at 4 °C. The protein content in the supernatant was determined using a Bio-Rad Protein Assay Kit (Bio-Rad, Hercules, CA, USA). Protein extracts were subjected to sodium dodecyl sulfate-polyacrylamide gel electrophoresis and blotted onto polyvinylidene fluoride membranes. The membranes were blocked with blocking buffer, and then incubated at 4 °C overnight with primary antibodies against IRβ, CD36, and FFA synthase (1:200 dilution), and against PI3 kinase p85, phospho-p70 S6K (Thr389), p70 S6K, phospho-4E-BP1 (Thr37/46), 4E-BP1, phospho-AMPKα (Thr172), AMPKα, phospho-acetyl-CoA carboxylase (Ser79), acetyl CoA carboxylase, and acetyl CoA carboxylase 1 (1:1000 dilution). Primary mouse anti-IRS2 and anti-GAPDH antibodies were used at dilutions of 1:1000 and 1:3000, respectively. We visualized the blots by chemiluminescence after incubating with donkey anti-rabbit IgG or sheep anti-mouse IgG conjugated to horseradish peroxidase (1:2500). The immunoreactive bands were exposed and the signals were quantified using a cooled charge-coupled device camera system (LAS-3000 Mini; Fujifilm, Kanagawa, Japan).

### Very-low-density lipoprotein excretion test

Male Wistar rats were fed a casein control diet between 10:00 and 18:00 for 7 d prior to the experiment. After habituation, the rats (7.5 weeks of age, 208–229 g) were assigned to the 15PAA (*n* = 8), 5PAA (*n* = 8), and low Arg (*n* = 9) groups. The diet compositions are shown in Tables 1 and 2. The experimental diets were provided for 5 h from 9:00 to 14:00. One hour after removing the diets, we administered tyloxapol (200 mg/kg, dissolved in 0.9% NaCl; Triton WR-1339, Sigma-Aldrich, St. Louis, MO, USA) to all rats under isoflurane anesthesia (3–4%, 5 l/min; Dainippon Sumitomo Pharma, Osaka, Japan) via the tail vein. Blood was collected from the tail vein into chilled, heparinized tubes prior to tyloxapol injection and 30, 60, 120, and 240 min after injection to measure plasma TG concentrations. Tyloxapol inhibits endogenous lipoprotein lipase and blocks the clearance of lipid-carrying lipoproteins in the blood [19–21]. Thus, the rise in plasma TG concentration after tyloxapol injection is approximately proportional to the very-low-density lipoprotein (VLDL)-TG level. The TG concentrations in the plasma were measured with a commercial kit as described in “*Lipid extraction and TG measurements*.” The TG secretion rate was expressed in mg TG/dl/min.

### Respiratory exchange ratio

Six-week-old male Wistar rats were assigned to the 15PAA (*n* = 6), 5PAA (*n* = 6), and low Arg (*n* = 6) groups. Six days after initiation of the experimental diets, animals were individually placed in a metabolic chamber for 24 h, and VO_2_ and VCO_2_ were monitored every 10 min with an OXYMAX system (Columbus Instruments, Columbus, OH, USA). Rats were allowed free access to water and food during the experiment.

Theoretically, a respiratory exchange rate (RER) of 1.0 represents the dominant consumption of carbohydrates, and a decrease in the RER to approximately 0.7 represents proportionally higher lipid consumption.

### De novo lipogenesis assay

De novo lipogenesis was measured based on the method described previously with minor modifications [22]. Six-week-old male Wistar rats were assigned to the 15PAA (*n* = 8), 5PAA (*n* = 8), and low Arg (*n* = 8) groups (240–280 g) for 1 night (from 18:00 to 10:00). Rats were allowed free access to food. The following day, the rats of each group were further divided into D_2_O (Sigma Aldrich, St. Louis, MO, USA) or H_2_O injection groups (5 ml/kg body weight, intraperitoneally). Liver samples were collected under isoflurane anesthesia (3–4%, 5 l/min; Dainippon Sumitomo Pharma, Osaka, Japan) 24 h after injection and stored at − 80 °C until analysis. Lipids were extracted as described in “*Lipid extraction and TG measurements*” [18]. The extracted lipids were hydrolyzed and methylated with an FFA Methylation Kit (Nacalai Tesque, Kyoto, Japan), and the obtained products were purified with a Fatty Acid Methyl Ester Purification Kit (Nacalai Tesque). Purified FFA esters were analyzed by gas chromatography–mass spectrometry (GCMS-QP2010 Plus, SHIMADZU, Kyoto, Japan) to quantify palmitate isotopomers. The relative amounts of fatty acids were normalized by tissue weight and the difference in the total amount of methyl palmitate isotopomers in the D_2_O-injected group and H_2_O-injected group was interpreted as the de novo lipogenesis rate.

### Hepatic FFA uptake assay

We performed an in vivo FFA uptake assay according to the method published in previous reports [23, 24]. Six-week-old male Wistar rats were assigned to the 15PAA (*n* = 8), 5PAA (*n* = 8), and low Arg (*n* = 8) groups (240–280 g) for 1 night (from 18:00 to 10:00). The following day, the rats of each group were further divided into 2 groups; one was treated with fluorescent FFA analog (0.1 mg/kg, BODIPY® FL C_12_, Invitrogen, Carlsbad, CA, USA) and the other was treated with only vehicle (0.1% bovine serum albumin in phosphate-buffered saline) by intravenous injection under isoflurane anesthesia (3–4%, 5 l/min; Dainippon Sumitomo Pharma, Osaka, Japan). The liver samples were collected 10 min after injection and stored at − 80 °C until analysis. Lipids were extracted [18] and reconstituted in isopropanol. Then, lipid fluorescence was measured with an ARVO X3 microplate reader (PerkinElmer, Waltham, MA, USA). All values were normalized by tissue weight, and the value of the vehicle-administered group was subtracted as background fluorescence from that of the BODIPY-administered group.

### Statistical analysis

All data are presented as the mean ± standard error. The data were statistically analyzed with the Ekuseru-Toukei 2010 software package (Social Survey Research Information, Tokyo, Japan). Statistical significance was calculated using one-way analysis of variance (ANOVA) with the Bonferroni and Tukey–Kramer post-hoc tests to assess differences between groups. *P* < 0.05 was considered statistically significant.

## Results

### Diet-induced changes in the body composition of rats

**Fig. 1:**
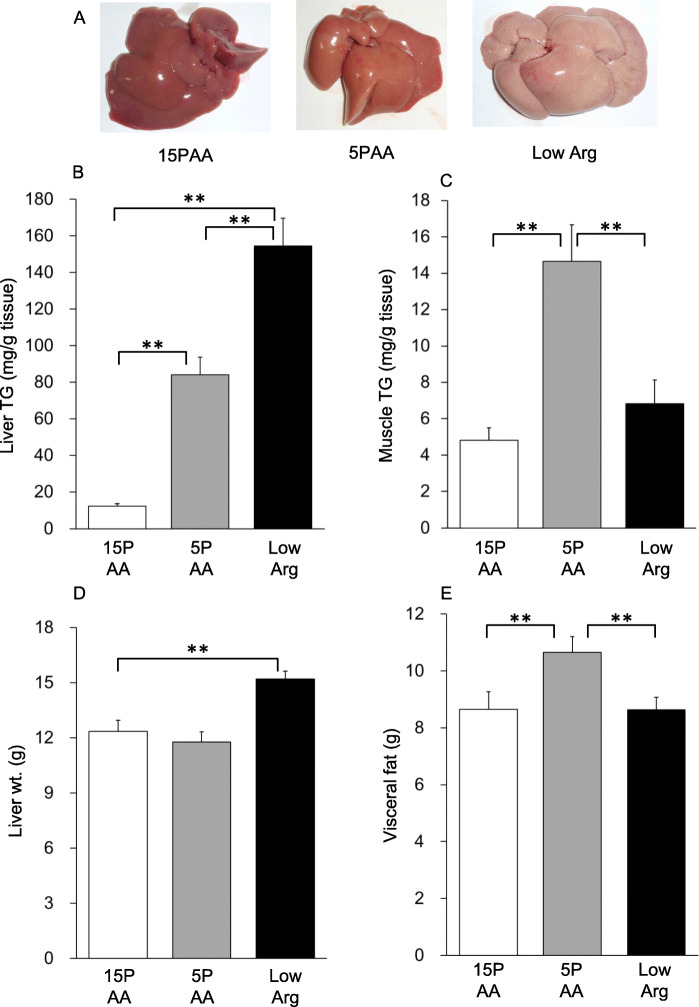
Effects of the 5PAA and low Arg diets on the liver. Macroscopic liver characteristics (**a**), hepatic (**b)** and longissimus muscle (**c**) TG contents, liver weight (**d**), and visceral fat weight (**e**) in 6-week-old male Wistar rats fed the 15PAA (*n* = 8), low Arg (*n* = 8), and 5PAA (*n* = 8) diets for 14 d. The values are means ± standard errors. Asterisks indicate significant differences as assessed by one-way ANOVA with Bonferroni and Tukey–Kramer post-hoc tests (*p < 0.05; **p < 0.01)

Body weight gain was slightly lower in the 5PAA group than in the 15PAA group, whereas the weight gain of the low Arg group was comparable to that of the 15PAA group (Table 3). Liver TG levels were significantly higher in the 5PAA group than in the 15PAA group (Fig. 1b, *p* < 0.01) as previously reported [9, 16]; however, even higher TG levels were observed in the low Arg group than in the 5PAA group (Fig. 1b, *p* < 0.01 vs. 15PAA and 5PAA). Macroscopic observations also supported this result; the livers of the 5PAA and low Arg groups were lighter in color than those of the 15PAA group (Fig. 1a). The TG content in the longissimus muscle and the visceral fat volume were significantly higher in the 5PAA group compared to the 15PAA group, whereas these parameters were not affected by the low Arg diet (Fig. 1c, e). Liver weight was significantly higher in the low Arg group than the 15PAA group, but this was not the case for the 5PAA group (Fig. 1d). These data suggested that, in contrast to our expectation, a low-total-amino acid diet and low-arginine diet might lead to different phenotypes, given that the low Arg diet did not completely recapitulate the effects of 5PAA administration.

**Table 3:** Blood biochemical parameters in the postprandial condition after feeding the low-protein and low-arginine diets for 14 days

	15PAA	5PAA	Low Arg
n	8	8	9
Body weight (g)	286 +/− 5 ^a^	267 +/− 5 ^b^	283 +/− 5 ^a b^
Total cholesterol (mg/dl)	69.9 +/− 2.8^a^	65.0 +/− 2.0^a^	50.8 +/− 2.3^b^
HDL cholesterol (mg/dl)	14.8 +/− 0.6	11.6 +/− 0.7	13.7 +/− 0.5
Glucose (mg/dl)	202 +/− 8	196 +/− 9	209 +/− 9
Insulin (ng/ml)	3.2 +/− 0.2^ab^	1.9 +/− 0.4^a^	4.6 +/− 0.6^b^

Data are shown as the mean +/− S.E.M.

Different superscript letters denote significant differences (*p* < 0.05)

### Diet-induced changes in blood biochemical parameters

**Fig. 2:**
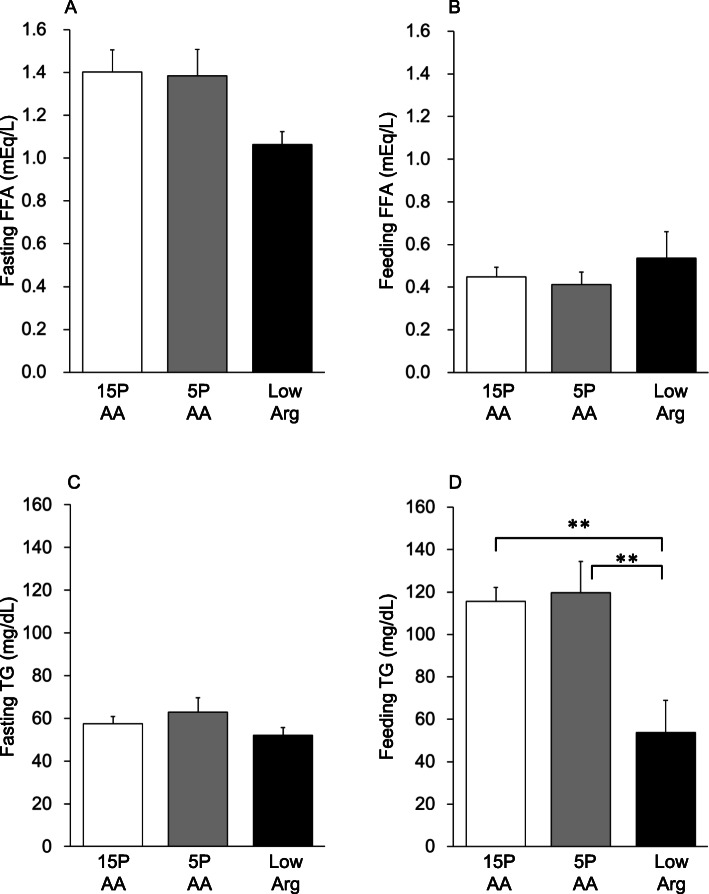
Plasma free fatty acid and TG levels in preprandial or postprandial conditions after feeding the 5PAA and low Arg diets. Six-week-old male Wistar rats were fed the 15PAA (*n* = 8), low Arg (*n* = 8), and 5PAA (*n* = 8) diet for 14 d. Preprandial free fatty acid (**a**) and TG (**c**) levels and postprandial free fatty acid (**b**) and TG (**d**) levels are shown. The values are the means ± standard errors. Asterisks indicate significant differences as assessed by one-way ANOVA with Bonferroni and Tukey–Kramer post hoc tests (**p < 0.01)

The blood glucose and insulin levels were over 190 mg/dl and 1.9 ng/ml, respectively, in the postprandial blood (Table 3, Supplementary Table 1). No differences were observed in the FFA concentrations of the fasting and postprandial blood among the 3 groups (Fig. 2a and b). By contrast, postprandial plasma TG and total cholesterol levels were significantly lower in the low Arg group than in the 15PAA group (Fig. 2d, Table 3), whereas the 5PAA diet did not affect plasma TG levels of the preprandial and postprandial blood (Fig. 2c and d). The high-density lipoprotein cholesterol concentrations were similar among the 3 groups in the preprandial and postprandial blood (Table 3).

### Glucose tolerance

**Fig. 3:**
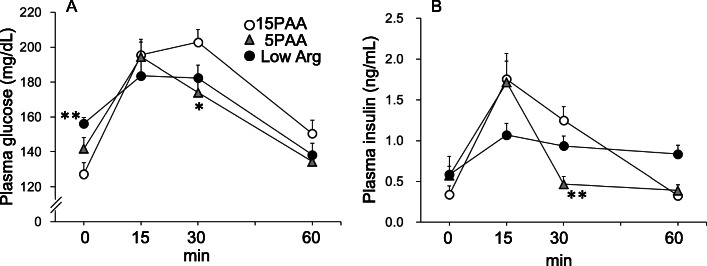
Effects of the 5PAA and low Arg diets on blood glucose and insulin levels after glucose loading. Six-week-old male Wistar rats were fed the 15PAA (n = 8), low Arg (*n* = 8), and 5PAA (*n* = 8) diets, and then OGTTs were performed on d 12 after initiation of the experimental diets. Plasma glucose (**a**) and insulin (**b**) concentrations are shown. The values are the means ± standard errors. Asterisks indicate significant differences as assessed by one-way ANOVA with Bonferroni and Tukey–Kramer post-hoc tests (*p < 0.05; **p < 0.01)

When the OGTT was performed at 12 d after initiation of the experimental diets, the plasma glucose levels of the 5PAA group were lower than those of the 15PAA group at 30 and 60 min after glucose loading (Fig. 3a). In addition, the plasma insulin levels were significantly lower in the 5PAA group than in the 15PAA group at 30 min after glucose loading (Fig. 3b). The fasting plasma glucose levels in the low Arg group were higher than in the 15PAA group (Fig. 3a), whereas the fasting plasma insulin levels were not significantly different between the low Arg and 15PAA groups (Fig. 3b). The plasma glucose level after glucose loading was not affected by the low Arg diet. The plasma insulin levels increased less in the low Arg group after glucose loading than in the 5PAA and 15PAA groups. The plasma insulin levels fell at 60 min after glucose loading in the 15PAA group but did not decrease in the low Arg group.

### Hepatic insulin receptor signaling

**Fig. 4:**
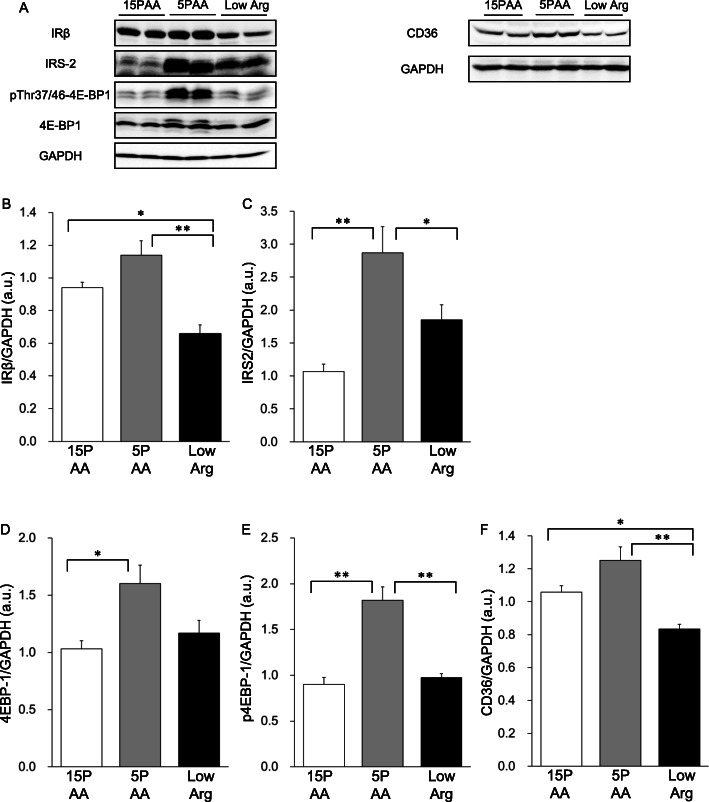
Insulin signaling-related protein levels in rats fed diets low in protein or arginine. Six-week-old male Wistar rats were fed the 15PAA (*n* = 8), low Arg (*n* = 8), and 5PAA (*n* = 8) diets for 14 d. Whole-liver lysates were analyzed by immunoprecipitation and immunoblotting with antibodies against IRβ (**b**), IRS-2 (**c**), 4E-BP1 (**d**), phosphorylated 4E-BP1 (Thr37/46) (E), CD36 (**f**), and GAPDH as an internal control. Representative immunoblots (**a**) are shown. The values are the means ± standard errors. Asterisks indicate significant differences as assessed by one-way ANOVA with Bonferroni and Tukey–Kramer post-hoc tests (*p < 0.05; **p < 0.01)

Western blotting analysis revealed that the expression of IRβ was slightly, but not significantly, higher in the livers of 5PAA-fed rats than those of the 15PAA-fed rats (Fig. 4a and b). Rats fed the 5PAA diet exhibited markedly higher expression levels of IRS2 (Fig. 4a and c) in the liver than rats fed the 15PAA or low Arg diet. Expression of the downstream effector 4E-BP1 (Fig. 4a and d) and its threonine phosphorylation were significantly higher in the 5PAA group than in the 15PAA or low Arg group (Fig. 4a and e). In contrast, low Arg-fed rats showed a lower total IRβ level than the 15PAA- and 5PAA-fed rats, and IRS2, 4E-BP1, and phosphorylated 4E-BP1 levels were comparable to those of 15PAA-fed rats. These results further indicated that the 5PAA and low Arg diets had disparate effects on liver insulin signaling.

### TG secretion, de novo lipogenesis, FFA uptake, and energy consumption

**Fig. 5:**
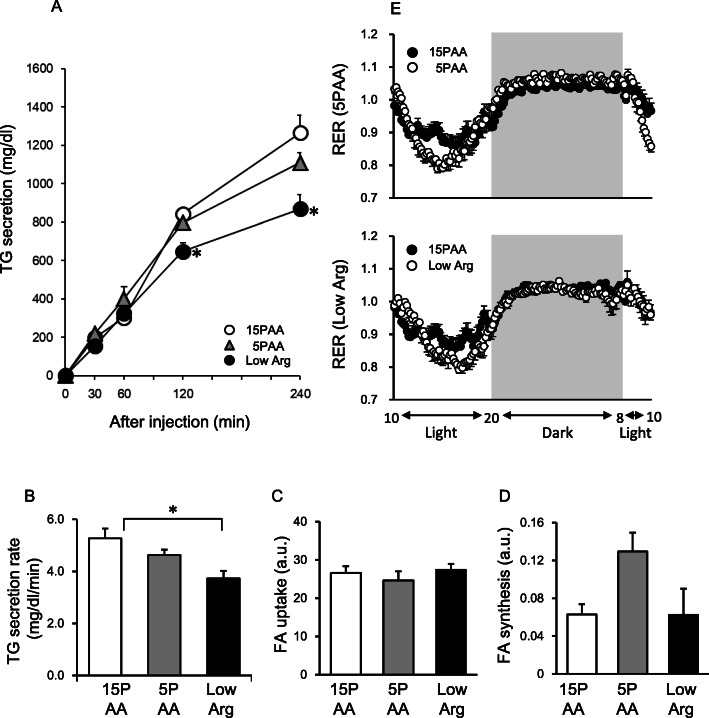
VLDL secretion from the livers, lipogenesis, fatty acid uptake, and RERs of rats fed the 5PAA and low Arg diets. Rats were fed the 15PAA (n = 8), 5PAA (*n* = 8), and low Arg (*n* = 8) diets. After 5 h of feeding, rats were injected with tyloxapol (200 mg/kg), and then TG concentrations (**a** and **c**) were measured at 0, 30, 60, 120, and 240 min post-injection. The RER (**b**) of each rat (*n* = 6) was measured for 24 h. A de novo lipogenesis assay (**d**) was performed by measuring palmitate isotopomers after intraperitoneal injection of D_2_O or H_2_O into rats fed the 15PAA (*n* = 8), 5PAA (*n* = 8), and low Arg (*n* = 8) diets for 1 night (18:00–10:00). Fatty acid uptake (**e**) was evaluated by incorporation of a fatty acid analog, BODIPY® FL C12, into the livers of rats fed the 15PAA (*n* = 4), 5PAA (*n* = 4), and low Arg (*n* = 4) diets for 1 night (18:00–10:00), and fluorescence was measured in the liver lipid extractions. The values are the means ± standard errors. Asterisks indicate significant differences assessed by one-way ANOVA with Bonferroni and Tukey–Kramer post-hoc tests (*p < 0.05)

We next assessed hepatic lipid flux in rats fed the 5PAA and low Arg diets. For the hepatic TG secretion assay, the rats were fed the indicated experimental diets for 5 h after a 15-h fast, and then tyloxapol was injected into the tail vein. The rats in the 3 groups consumed approximately equal amounts of the diets during the 5 h of feeding (15PAA: 13 ± 1 g; 5PAA: 14 ± 1 g; and low Arg: 14 ± 0 g). When plasma TG concentrations were measured at 0, 30, 60, 120, and 240 min after tyloxapol injection, every group showed time-dependent increases, but the rate of increase of the low Arg group was significantly lower than that of the 15PAA group (Fig. 5a). Remarkably, the hepatic TG secretion rate of the 5PAA group did not significantly change compared to that of the 15PAA group (Fig. 5b). Analysis of hepatic FFA uptake activity using a fluorescent FFA analog showed comparable levels among the 3 groups, although the expression of CD36 in the liver, which mediates cellular FFA uptake, was significantly lower in the low Arg group than in the other groups (Fig. 4a and f, c). In addition, the de novo lipogenesis assay demonstrated that the lipid synthetic activity in the liver was enhanced only in the 5PAA group, whereas it was unchanged in the low Arg group (Fig. 5d).

We also measured the RER of the 3 groups (Fig. 5e, Supplementary Figure 2). RER is a stoichiometric ratio of exhaled CO_2_ to inhaled O_2_, which roughly reflects the oxidized energy source. The RERs of the 3 groups measured during the night were almost comparable at around 1.0, whereas the RERs measured during the day were slightly lower in the 5PAA and low Arg groups than in the 15PAA group, indicating that the 5PAA diet and low Arg diet did not slow lipid consumption in comparison with the 15PAA diet.

### Hepatic TG secretion-related gene expression in the liver

We performed microarray analysis of liver samples from the rats of the 3 groups to evaluate the effects of the diets on hepatic transcripts (Supplementary Table 2). The results showed a marked decrease (0.47-fold) in the expression of the *ApoA4* gene in the livers of the low Arg group compared with that of the 15PAA livers, which was validated by quantitative PCR analysis. ApoA4, an apolipoprotein primarily expressed in the intestine in mammals, is believed to be involved in chylomicron formation [25]. However, in rodents, it is reportedly also expressed in the liver where it is associated with VLDL formation and TG secretion [20].

**Fig. 6:**
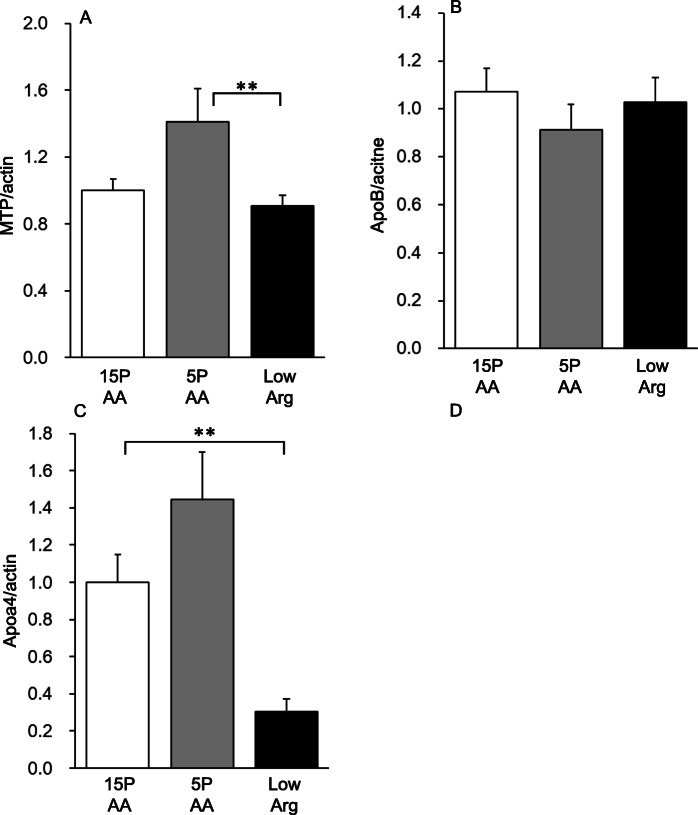
Effects of the 5PAA and low Arg diets on the expression of VLDL assembly-related genes. Six-week-old male Wistar rats were fed the 15PAA (*n* = 8), low Arg (*n* = 8), and 5PAA (*n* = 8) diets for 14 d. Total RNA was isolated from the liver and then subjected to quantitative RT-PCR. The transcript expression levels for *Mttp* (**a**), *ApoB* (**b**), and *ApoA4* (**c**) are shown. Asterisks indicate significant differences as assessed by one-way ANOVA with Bonferroni and Tukey–Kramer post-hoc tests (**p < 0.01)

There were no significant differences in *ApoB* and *Mttp* mRNA levels, which are also important genes for hepatic VLDL formation, between the 15PAA and low Arg groups (Fig. 6a, b), whereas there was a tendency of *Mttp* mRNA levels to be slightly increased in the livers of the rats in the 5PAA group (Fig. 6A, *p* < 0.08 vs. 15PAA). However, the low Arg diet caused a dramatic decrease in *ApoA4* mRNA levels compared with the 15PAA diet, whereas the 5PAA diet had no effect (Fig. 6c).

## Discussion

We previously reported that both dietary total amino acid deficiency and selective arginine deficiency caused fatty liver, and activation of insulin receptor signaling is known to stimulate lipid accumulation [9, 16]. Therefore, we speculated that the phenotypes caused by a low Arg diet simply reflected those caused by the 5PAA diet, which could be mediated through enhanced insulin sensitivity. However, in the present study, we identified several distinct phenotypes in the 5PAA and low Arg groups, including the lipid accumulation level, serum lipid profile, glucose tolerance, and insulin signal activities. In addition, because liver TG content is thought to be determined by the integration of de novo lipogenesis, TG secretion, FFA uptake, and lipid oxidation [26], we evaluated these activities in rats fed the 5PAA or low Arg diet. Contrary to our expectations, these parameters also completely differed between the groups; only the 5PAA group exhibited increased lipogenesis, only the low Arg group showed attenuated TG secretion, and FFA uptake and RER scarcely differed among the groups. The RER was slightly lower, particularly during the day, in the 5PAA and low Arg groups than in the 15PAA group, which indicated that the lipid oxidation rates of the experimental groups were almost the same or slightly higher than that of the 15PAA group. However, as the RER indicates the opposite of lipid accumulation, it cannot be responsible for fatty liver development. Collectively, these results indicate that distinct mechanisms underlie the fatty liver formation induced by total amino acid deficiency and only arginine deficiency.

IRβ and IRS2 are pivotal mediators of insulin activity in the liver. We found that the 5PAA diet significantly increased the protein expression levels of total IRS2 and 4EBP1 and 4EBP1 phosphorylation (*p* < 0.05 in comparison with 15PAA), and tended to increase total IRβ expression in the liver. This suggests that the 5PAA diet may potentiate insulin signaling. The activation of this pathway is known to upregulate FFA synthesis [9, 27–29], and, indeed, increased de novo lipogenesis was observed in 5PAA diet-fed rats. In contrast, we recently reported that when hepatocytes were cultured in an amino acid-sufficient or -deficient medium, the intracellular TG level was increased by amino acid deficiency without the addition of any lipids or hormones, and was accompanied by enhanced lipid synthesis [10]. These results indicate that hepatocytes themselves monitor extracellular amino acid concentrations to induce lipid accumulation in a cell-autonomous manner. It is possible that both the lipogenesis enhanced by insulin signal potentiation and that stimulated directly by amino acid deficiency may lead to lipid accumulation in the liver in response to 5PAA feeding. On the other hand, the low Arg diet decreased IRβ and did not have any statistically significant effects on IRS2 and 4E-BP1 protein levels. These findings support the conclusion that the 5PAA and low Arg diets induced hepatic fat accumulation via different mechanisms. 5PAA diet-induced TG accumulation appears to be mediated through insulin signaling, whereas low Arg diet-induced accumulation was insulin-independent.

We also demonstrated that only the 5PAA diet induced visceral and muscular fat accumulation, in addition to accumulation in the liver. Some reports have shown that a low-protein diet leads to intramuscular and other fat deposition in piglets [30–32], and a high-protein diet reduces visceral fat and hepatic TG levels in humans [2, 33]. In addition, we previously found that the activation of insulin signaling in response to consumption of the 5PAA diet was specific to the liver and was not observed in the white adipose tissue or muscle [9]. Taken together, these results suggest that the dietary protein content and amino acid balance can provoke a tissue-specific reaction, and are, thus, important determinants of body fat deposition in mammals.

The assembly and secretion of VLDL are the key regulatory steps of plasma and intrahepatic TG and FFA levels owing to its glyceride-rich structure, and are closely linked to the progression of NAFLD. The 5PAA diet had no effect on postprandial plasma TG and fasting FFA levels, whereas the low Arg group had lower postprandial plasma TG and liver CD36 levels than the control group, although their fasting FFA levels were comparable. By contrast, neither the 5PAA nor low Arg diet influenced FFA uptake by the liver. The scavenger FFA receptor CD36 is known to mediate FFA uptake into the metabolic tissues, and is reportedly associated with non-alcoholic fatty liver development [23, 34]. However, in our model, changes in the hepatic CD36 level and blood TG/FFA levels did not affect FFA uptake into the liver, suggesting that the change in serum lipid concentrations was unlikely to be a direct reason for fatty liver formation.

In this study, we analyzed the VLDL secretion rate after tyloxapol injection. Tyloxapol is a non-ionic detergent that prevents the catabolism of triacylglycerol-rich lipoproteins by lipoprotein lipase [19, 35]; it is widely used for the in vivo determination of the secretion and clearance rates of VLDL [21]. We found that VLDL secretion in the 5PAA group was not significantly different to that in the 15PAA group, whereas that of the low Arg group was significantly lower. These findings suggested that the hepatic lipid accumulation caused by the low Arg diet, but not the 5PAA diet, was attributable to the downregulation of VLDL secretion. As the inhibition of VLDL secretion was observed within just 5 h of the initiation of the low Arg diet, it may be the major cause of fatty liver formation induced by the low Arg diet.

Although *Mttp* and *ApoB* are the most important elements in VLDL assembly [36], neither the 5PAA nor the low Arg diet statistically affected the expression of these genes. Instead, we discovered a decrease in the expression level of *ApoA4*. ApoA4, a 46-kDa protein with a lipid-binding site, is expressed in the intestine and liver of rodents [25]. It is generally believed to be expressed primarily in the small intestine in humans, although minor expression in the human liver and human hepatocyte lines has also been reported [37, 38]. Adenoviral overexpression of ApoA4 in the liver increases VLDL secretion and decreases hepatic TG levels, and *ApoA4*-knockout mice show lower VLDL secretion than wild-type controls [20]. Thus, ApoA4 is another important apolipoprotein for VLDL formation. Based on these findings, we believe that the downregulation of ApoA4 expression in response to the low Arg diet may attenuate VLDL secretion.

Arginine and lysine use the same membrane transporter system. Lysine is an important amino acid for growth and nutritional metabolism. It tends to be the first limiting amino acid in a variety of circumstances. Low dietary lysine in piglets drives intramuscular fat accumulation and a reduction in serum glucose levels in growing pigs [39]. In our previous study, however, feeding rats a low Arg diet did not affect serum arginine and lysine concentrations, which was presumably due to the compensatory synthesis of arginine. On the other hand, the serum levels of other amino acids, such as methionine and histidine, were significantly increased by a low Arg diet [10]. Therefore we do not consider arginine alone a signal molecule. Instead, we believe that the complex alteration of the comprehensive serum amino acid profile is important. In support of that hypothesis, in the same previous report, a nonlinear analysis based on a mathematical machine learning method revealed that the comprehensive amino acid profile correlated with TG levels in the liver although no single amino acid concentration correlated well. The molecular basis of how such information is sensed by cells remains unclear. We are currently investigating the molecular sensing mechanism and preparing a report on that topic.

We demonstrated that a mild deficiency in dietary total amino acids induces fat accumulation in visceral and ectopic, but not subcutaneous, fat. Increases in ectopic fat accumulation and intra-abdominal fat are thought to occur in parallel [40, 41]. Feeding a low-protein diet to piglets leads to the deposition of intramuscular and other fat [30, 31]. In healthy humans, a high-protein diet reduces visceral fat [2], and a high-protein/low-carbohydrate diet also lowers hepatic TG levels [33]. Therefore, dietary protein content is an important determinant of body fat deposition in mammals.

## Conclusions

Our present results demonstrated that a mild deficiency in dietary total amino acids or solely in dietary arginine caused a large increase in TG accumulation in the liver via different mechanisms, although both diets lacked arginine to the same extent. The low Arg diet caused hepatic lipid accumulation by attenuating VLDL secretion, which may be caused, at least in part, by the downregulation of ApoA4 expression. Although the concrete molecular mechanisms remain elusive, understanding how these differences come about may explain the tissue-specific responses to dietary protein malnutrition with regard to insulin-like activity and lipid metabolism.

## Supplementary information

**Additional file 1: Figure S1** Insulin signaling-related protein levels in rats fed the 5PAA and low Arg diets. Six-week-old male Wistar rats were fed the 15PAA (*n* = 8), low Arg (*n* = 8), or 5PAA (*n* = 8) diet for 14 d. Whole-liver lysates were analyzed by immunoprecipitation and immunoblotting with antibodies against p85 (B), S6K (C), pS6K (D), AMPK (F), and pAMPK (G), where anti-S6K served as the internal control; or with antibodies against pS6K/S6K (E) and pAMPK (H), where anti-GAPDH served as the internal control. **Figure S2** Average RER values during the light period (8:00–20:00) and dark period (20:00–8:00) were calculated individually.

**Additional file 2: Table S1** Plasma glucose and insulin levels in rats fed the 5PAA and low Arg diets. **Table S2** List of apolipoprotein genes.

## Data Availability

The datasets used and/or analyzed during the current study are available from the corresponding author upon reasonable request.

## Abbreviations

15PAA: Diet containing amino acids from casein equivalent to 15% protein
4E-BP1: Eukaryotic initiation factor 4E-binding protein
5PAA: Diet containing amino acids from casein equivalent to 5% protein
ANOVA: Analysis of variance
ApoA4: Apolipoprotein A-IV
ApoB: Apolipoprotein B
FFA: Free fatty acid
IRβ: Insulin receptor subunit β
IRS2: Insulin receptor substrate 2
MTP: Microsomal triglyceride transfer protein
NAFLD: Non-alcoholic fatty liver disease
OGTT: Oral glucose tolerance test
PCR: Polymerase chain reaction
RER: Respiration exchange rate
RT: Reverse transcription
S6K: S6 kinase
TG: Triglyceride
VLDL: Very-low-density lipoprotein
