# Supplementary material for: Low-arginine and low-protein diets induce hepatic lipid accumulation through different mechanisms in growing rats
Source: Nutr Metab (Lond). 2020 Aug 3;17:60. doi: 10.1186/s12986-020-00477-5 (PMC7398226; doi:10.1186/s12986-020-00477-5)
Supplement: Supplementary file 1 — Additional file 1: Figure S1 Insulin signaling-related protein levels in rats fed the 5PAA and low Arg diets. Six-week-old male Wistar rats were fed the 15PAA (n = 8), low Arg (n = 8), or 5PAA (n = 8) diet for 14 d. Whole-liver lysates were analyzed by immunoprecipitation and immunoblotting with antibodies against p85 (B), S6K (C), pS6K (D), AMPK (F), and pAMPK (G), where anti-S6K served as the internal control; or with antibodies against pS6K/S6K (E) and pAMPK (H), where anti-GAPDH served as the internal control. Figure S2 Average RER values during the light period (8:00–20:00) and dark period (20:00–8:00) were calculated individually. [file 12986_2020_477_MOESM1_ESM.pptx]

## Slide 1
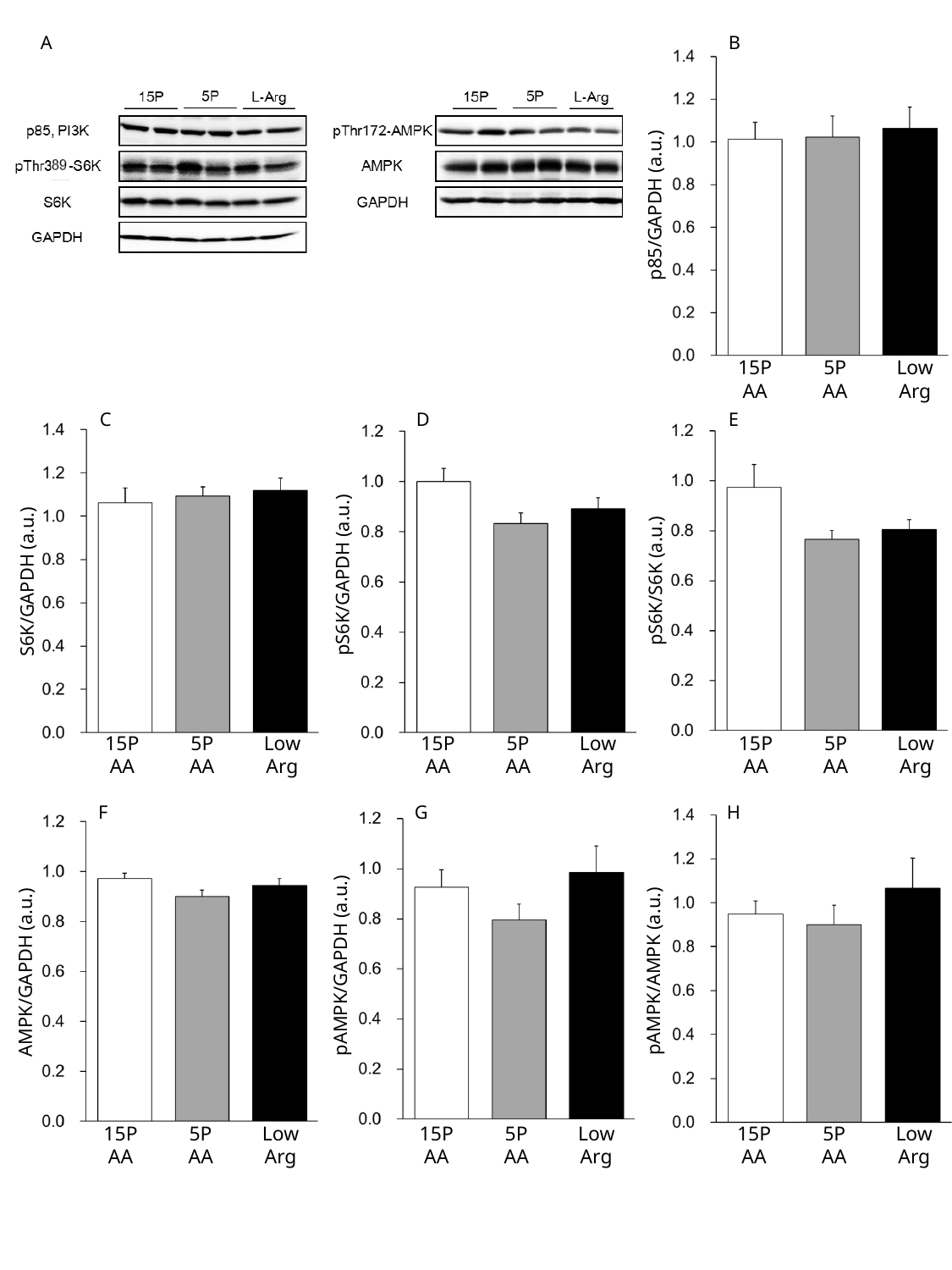

A
B
p85/GAPDH (a.u.)
15P
AA
5P
AA
Low
Arg
C
D
pS6K/GAPDH (a.u.)
S6K/GAPDH (a.u.)
15P
AA
5P
AA
Low
Arg
15P
AA
5P
AA
Low
Arg
E
pS6K/S6K (a.u.)
15P
AA
5P
AA
Low
Arg
F
G
H
pAMPK/GAPDH (a.u.)
pAMPK/AMPK (a.u.)
AMPK/GAPDH (a.u.)
15P
AA
5P
AA
Low
Arg
15P
AA
5P
AA
Low
Arg
15P
AA
5P
AA
Low
Arg

## Slide 2
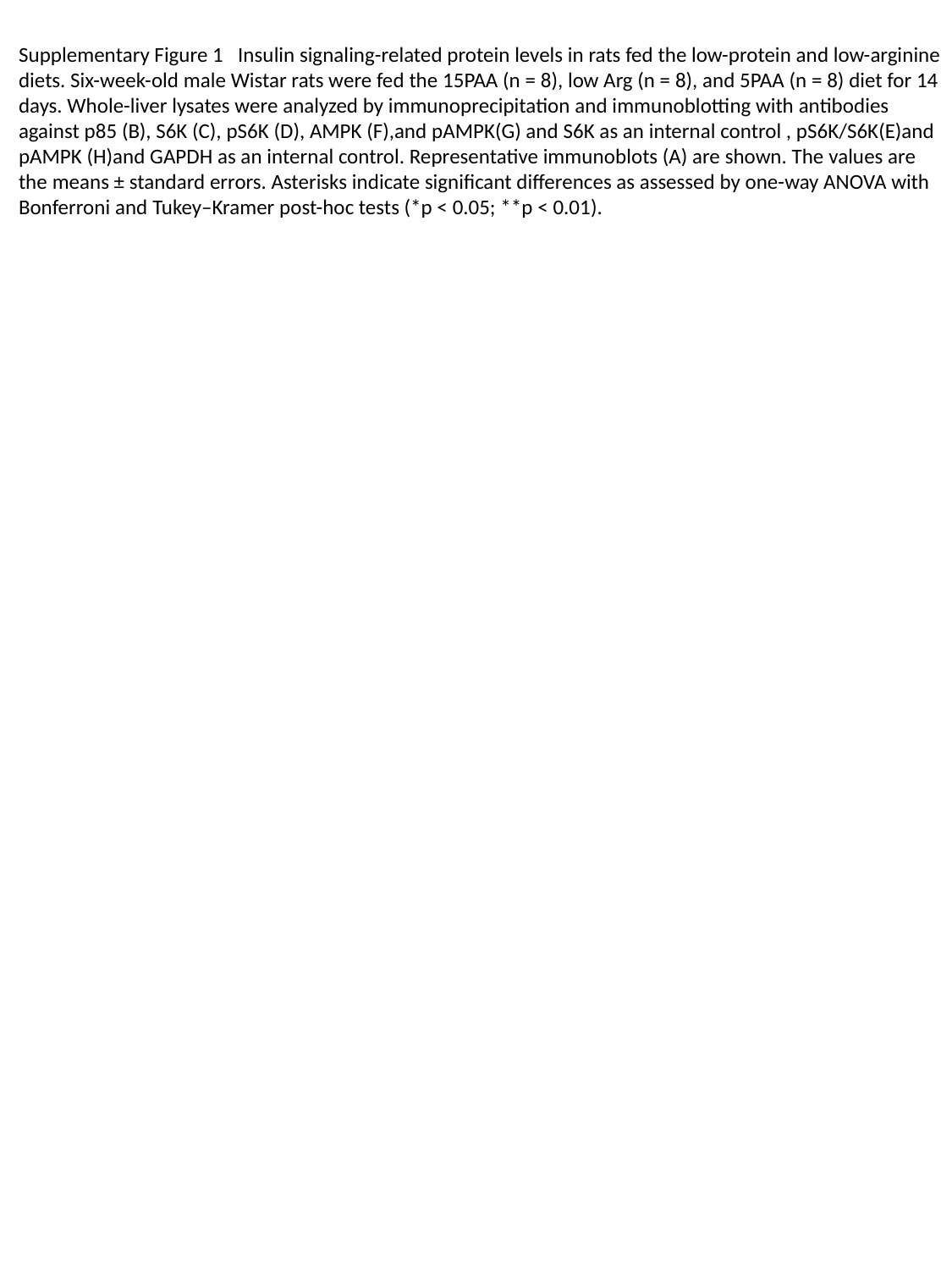

Supplementary Figure 1 Insulin signaling-related protein levels in rats fed the low-protein and low-arginine diets. Six-week-old male Wistar rats were fed the 15PAA (n = 8), low Arg (n = 8), and 5PAA (n = 8) diet for 14 days. Whole-liver lysates were analyzed by immunoprecipitation and immunoblotting with antibodies against p85 (B), S6K (C), pS6K (D), AMPK (F),and pAMPK(G) and S6K as an internal control , pS6K/S6K(E)and pAMPK (H)and GAPDH as an internal control. Representative immunoblots (A) are shown. The values are the means ± standard errors. Asterisks indicate significant differences as assessed by one-way ANOVA with Bonferroni and Tukey–Kramer post-hoc tests (*p < 0.05; **p < 0.01).

## Slide 3
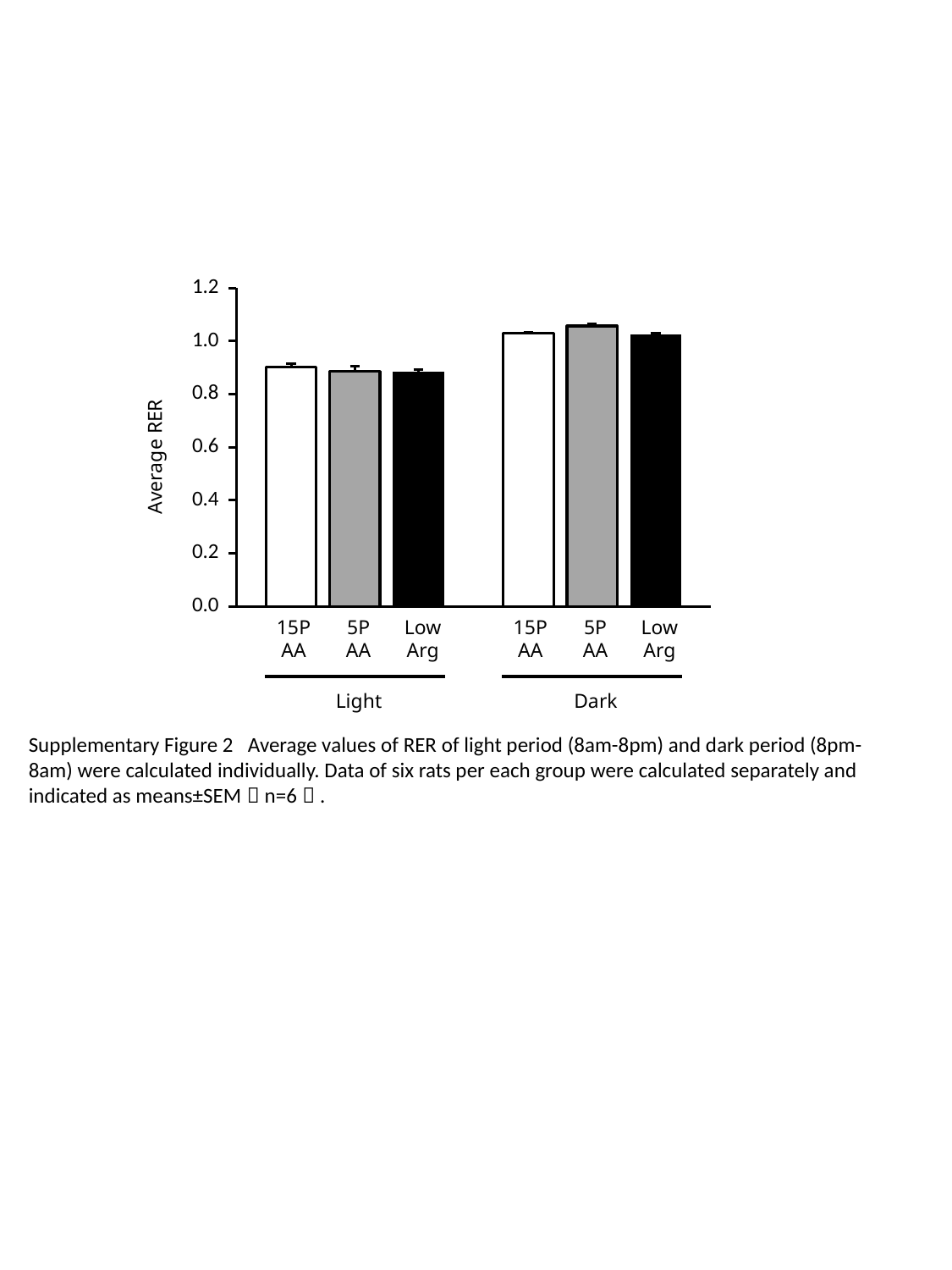

### Chart
| Category | | | |
|---|---|---|---|
| Day7 light | 0.903158016265344 | 0.8863169882320744 | 0.8830898170671778 |
| Day7 dark | 1.0310787037037035 | 1.0571273148148148 | 1.0222685185185185 |Average RER
15P
AA
5P
AA
Low
Arg
15P
AA
5P
AA
Low
Arg
Dark
Light
Supplementary Figure 2 Average values of RER of light period (8am-8pm) and dark period (8pm-8am) were calculated individually. Data of six rats per each group were calculated separately and indicated as means±SEM（n=6）.
